# Navigation Grade MEMS IMU for A Satellite

**DOI:** 10.3390/mi12020151

**Published:** 2021-02-04

**Authors:** Wanliang Zhao, Yuxiang Cheng, Sihan Zhao, Xiaomao Hu, Yijie Rong, Jie Duan, Jiawei Chen

**Affiliations:** Shanghai Aerospace Control Technology Institute, 1555 Zhongchun Road, Shanghai 201109, China; zhaodada999@163.com (W.Z.); ssyzxgzqtzskct@163.com (S.Z.); shandahu2008@163.com (X.H.); rongyijie@163.com (Y.R.); dj405007@163.com (J.D.); cjw1105802603@gmail.com (J.C.)

**Keywords:** IMU, MEMS gyroscopes, satellite, navigation grade, commercial aerospace

## Abstract

This paper presents a navigation grade micro-electromechanical system (MEMS) inertial measurement unit (IMU) that was successfully applied for the first time in the Lobster-Eye X-ray Satellite in July 2020. A six-axis MEMS gyroscope redundant configuration is adopted in the unit to improve the performance through mutual calibration of a set of two-axis gyroscopes in the same direction. In the paper, a satisfactory precision of the gyroscope is achieved by customized and self-calibration gyroscopes whose parameters are adjusted at the expense of bandwidth and dynamics. According to the in-orbit measured data, the MEMS IMU provides an outstanding precision of better than 0.02 °/h (1σ) with excellent bias instability of 0.006 °/h and angle random walk (ARW) of around 0.003 °/h^1/2^. It is the highest precision MEMS IMU for commercial aerospace use ever publicly reported in the world to date.

## 1. Introduction

In the past decades, considerable attention has been paid to micro-electromechanical system (MEMS) inertial devices, including gyroscopes and accelerometers owing to their small sizes, low cost, and high sensitivity [1]. After decades of technology development, MEMS gyroscopes with mid–low-end consumer grade and industrial grade precisions have been widely applied in automobiles, mobile phones, consumer electronics, and other fields [2,3,4,5,6].

However, in the high-end inertial application market, it is challenging for the current MEMS gyroscopes to compete with the fiber optic gyroscope in precision [7,8]. The precision of mainstream high-precision MEMS gyroscope on today’s market is usually around 0.5 °/h, such as Sensonor’s typical products STIM202 [9] and STIM210 [10]. The highest precision of Honeywell’s HG4930 [11] can reach 0.25 °/h, with angle random walk (ARW) of 0.04 °/h^1/2^, representing the top level of MEMS inertial measurement unit (IMU) commercial applications worldwide at the moment.

**Figure 1:**
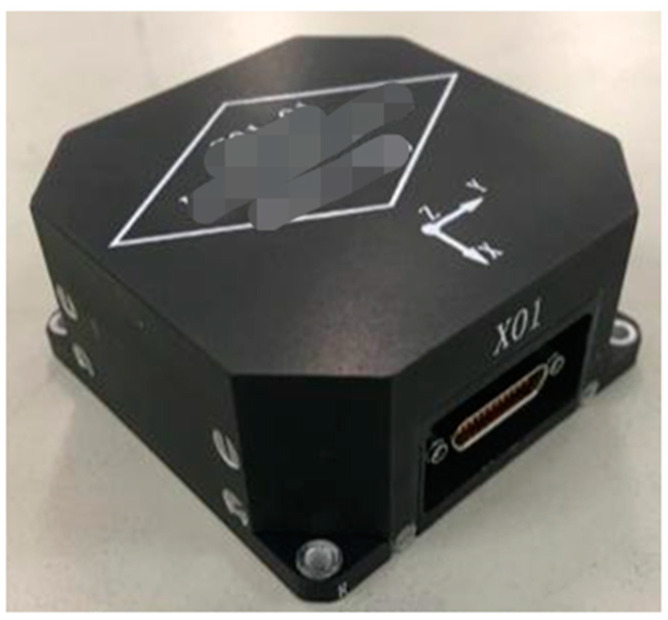
The navigation grade micro-electromechanical system (MEMS) gyroscope unit product.

On 25 July 2020, the Lobster-Eye X-ray Satellite (Einstein Probe Satellite), developed by China, for dark matter signal detection, was successfully launched [12]. A new high-precision MEMS IMU is carried out in the satellite’s attitude control system for the first time, as shown in Figure 1. This product has the bias stability of 0.02 °/h (1σ), bias instability of 0.006 °/h, AWR of about 0.003 °/h^1/2^, weight of less than 210 g, and power consumption of lower than 1.5 W, being the highest precision MEMS IMU product for the commercial aerospace application so far.

**Figure 2:**
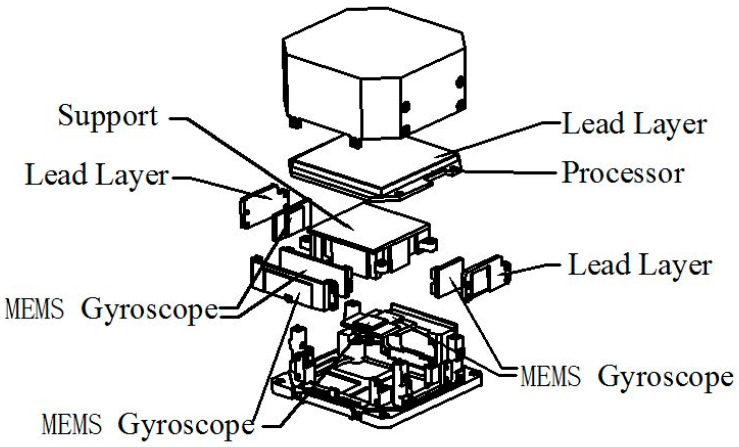
The structure diagram of the six-axis inertial measurement unit (IMU).

The MEMS IMU consists of six independent MEMS gyroscopes, processors for data acquisition and signal fusion processing, stress isolation support structure, lead layer for electromagnetic shielding and other parts, as schematically described in Figure 2. Each of the three orthogonal sensitive axes is equipped with two independent MEMS gyroscopes, which enhance the reliability and precision. In an effort to boost the product’s performance, the research mainly focused on the following three aspects of gyroscope design, combination environmental protection, and system-level calibration:

(1) For gyroscope design, the MEMS gyroscopes are manufactured using silicon-on-insulator (SOI) silicon technology, wafer-level stress isolation vacuum packaging, and matched with the self-calibrated application-specific integrated (ASIC) circuit. Furthermore, on account of the special environment of micro-nano satellite applications, the accuracy and noise index of MEMS gyroscopes are further promoted on the basis of meeting the requirements of satellite applications by sacrificing the bandwidth and measurement range.

(2) For MEMS IMU design, the external environment interference is largely investigated. Since the structure gap of the MEMS gyroscopes is very sensitive to stress variation, we exploit a dedicated support for gyroscope installation and implement a special low stress device installation method to reduce the influence of environmental stress such as vibration and temperature on the performance of gyroscopes [13]. In order to meet the radiation resistance requirements in space application, a protective lead layer is designed outside the gyroscope structure to isolate the gyroscopes device from the impact of space ions.

(3) For system-level calibration, the gyroscope noise can be lowered by the mutual calibration of two-axis MEMS gyroscopes with parallel redundant configuration in the same direction, and the information fusion of gyroscope angular rate output based on the Kalman filter.

## 2. Error Analysis

The high-precision MEMS gyroscope is the key point to the implementation of this IMU. In general, there are some factors in improving the MEMS gyroscope performance: (1) the stability of the resonator’s vibration amplitude, which could affect the scale factor of the gyroscope; (2) the orthogonality of the driving direction *X* and the sensitive direction *Y*, whose non-orthogonal term will lead to the drift of the gyroscope signal; (3) the asymmetry effect on the gyroscope arising from frequency split and damping split; (4) the change of resonator structure parameters caused by the environmental stress. 

After adding the control term, the dynamical model of the MEMS gyroscope with the non-idealities of frequency split and damping the split is as follows [14,15,16,17]:

(1) $$\left\{\begin{array}{l} \ddot{x}-2K\Omega \dot{y}+(\frac{2}{\tau }+\Delta (\frac{1}{\tau })\cos2\theta_{\tau })\dot{x}+\Delta (\frac{1}{\tau })\sin2\theta_{\tau }\dot{y}\\ +(\omega^{2}-\omega \Delta \omega \cos2\theta_{\omega })x-\omega \Delta \omega \sin2\theta_{\omega }y=f_{x}\\ \ddot{y}+2K\Omega \dot{x}+(\frac{2}{\tau }-\Delta (\frac{1}{\tau })\cos2\theta_{\tau })\dot{y}+\Delta (\frac{1}{\tau })\sin2\theta_{\tau }\dot{x}\\ +(\omega^{2}+\omega \Delta \omega \cos2\theta_{\omega })y-\omega \Delta \omega \sin2\theta_{\omega }x=f_{y} \end{array}\right.$$

where *f_x_,* and *f_y_* as the electrode forces imposed on the driving axis and the sensitive axis, which are controlled by driving direction and sensitive direction separately; *x* and *y* as the vibration displacements on the two vibration axes of the resonator; *τ* as the vibration attenuation constant of the resonator; *K* as the precession factor; *Ω* as the rotational speed; *ω* as the resonant frequency of the resonator; *θ_τ_* as the damping principal axis azimuth; and *θ_ω_* as the stiffness principal axis azimuth.

When the gyroscope is in the force feedback mode, we have:

(2) $$\left\{\begin{array}{l} x=A_{0}\sin\omega_{x}t\\ y=0 \end{array}\right.$$

That is to say, the driving direction *x* exhibits sinusoidal vibration with constant amplitude *A_0_* and vibration frequency *ω_x_* of the principal mode with the action of amplitude (AMP) control loop and the frequency tracking loop; The node amplitude of the sensing direction Y remains at 0 under the control of force to rebalance (FTR) loop. Putting the above solution into the dynamical model, it was extended to the following form:

(3) $$\left\{\begin{array}{l} -A_{0}\omega_{x}^{2}\sin\omega_{x}t+(\frac{2}{\tau }+\Delta (\frac{1}{\tau })\cos2\theta_{\tau })A_{0}\omega_{x}\cos\omega_{x}t+(\omega^{2}-\omega \Delta \omega \cos2\theta_{\omega })A_{0}\sin\omega_{x}t=f_{x}\\ 2K\Omega A_{0}\omega_{x}\cos\omega_{x}t+\Delta (\frac{1}{\tau })\sin2\theta_{\tau }A_{0}\omega_{x}\cos\omega_{x}t-\omega \Delta \omega \sin2\theta_{\omega }A_{0}\sin\omega_{x}t=f_{y} \end{array}\right.$$

By solving the above equation, it obtains:

(4) $$\left\{\begin{array}{l} \omega_{x}=\sqrt{\omega^{2}-\omega \Delta \omega \cos2\theta_{\omega }}\\ f_{x}=(\frac{2}{\tau }+\Delta (\frac{1}{\tau })\cos2\theta_{\tau })A_{0}\omega_{x}\cos\omega_{x}t\\ f_{y}=2K\Omega A_{0}\omega_{x}\cos\omega_{x}t+\Delta (\frac{1}{\tau })\sin2\theta_{\tau }A_{0}\omega_{x}\cos\omega_{x}t-\omega \Delta \omega \sin2\theta_{\omega }A_{0}\sin\omega_{x}t \end{array}\right.$$

where $(\frac{2}{\tau }+\Delta (\frac{1}{\tau })\cos2\theta_{\tau })A_{0}\omega_{x}$ represents the amplitude control term; $2K\Omega A_{0}\omega_{x}+\Delta (\frac{1}{\tau })\sin2\theta_{\tau }A_{0}\omega_{x}$ is the force equilibrium control term, which is also the gyroscope signal output; and $-\omega \Delta \omega \sin2\theta_{\omega }A_{0}$ denotes the orthogonal control term.

Then, we could state the output of the gyroscope signal:

(5) $$\begin{array}{cc} \Omega_{\mathrm{out}} & =\;K_{1}\cdot \Omega +B_{0}\\ & =\;2KA_{0}\omega_{x}\cdot \Omega +\Delta (\frac{1}{\tau })\sin2\theta_{\tau }A_{0}\omega_{x} \end{array}$$

To sum up, the scale factor of MEMS gyroscope is related to the resonant frequency. The slow drift of the resonant frequency would result in the drift of the scale factor, which would lower to the performance level of the gyroscope. Furthermore, the frequency split directly gives rise to the random drift of gyroscope, thus reducing the gyroscope precision. The existence of a frequency split would generate orthogonal errors on the resonator, causing the periodic traveling wave component to appear alternately in the standing wave. At this time, the standing wave position will swing periodically. Hence, for high-precision gyroscopes, the errors caused by frequency split should be restrained. In addition, as the MEMS chip is light in weight, the structure gap is very sensitive to the change of stress. Of course, the damping, noise and other environmental interference could also risk the gyroscope precision.

## 3. Design

### 3.1. Frequency Stabilization and Frequency Split Mitigation

**Figure 3:**
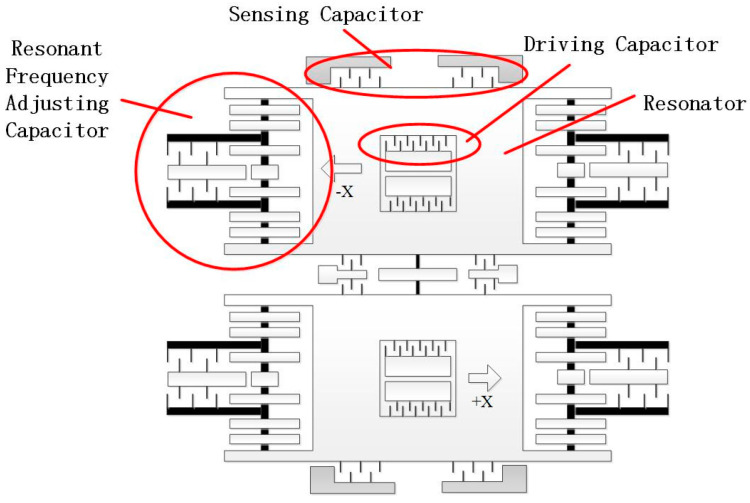
The structural design of the MEMS gyroscope.

It is known that the real-time tracking of resonant frequency could be realized by a phase-locked loop circuit [18], which, however, would still lead to the scale factor variation of the gyroscope [19]. In an effort to overcome this challenge, two movable mass blocks of the MEMS gyroscope are connected to the mass block anchor points of the two groups of driving frequency adjustment structures through the driving spring assembly in this paper. Taking advantage of the electrostatic negative stiffness effect, we form the driving frequency regulating capacitance through the movable electrode and fixed electrode structure, and undertake the closed-loop control of the gyroscope resonator’s stiffness, allowing the MEMS gyroscope resonant frequency to achieve closed-loop stability, as depicted in Figure 3.

In accordance with Formula (4), when the frequency split of the resonant frequencies occurs in the *X* and *Y* directions due to the structure asymmetry, the output of the gyroscope would drift. The larger the frequency split is, the greater the introduced drift is. 

**Figure 4:**
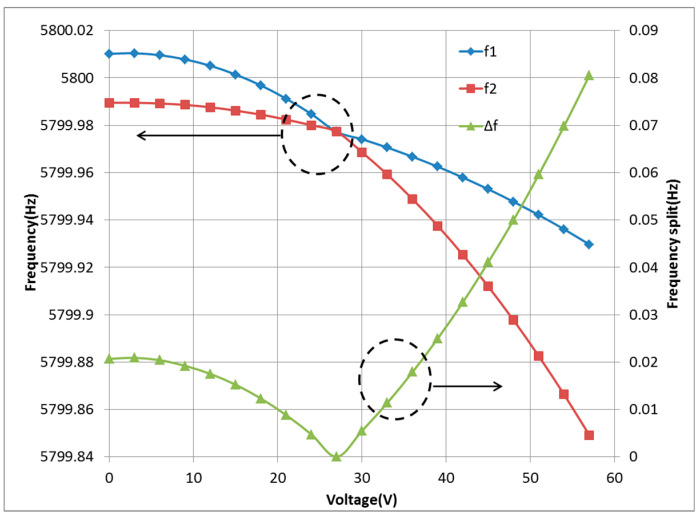
The result of multi-electrode electrical stiffness compensation.

In order to reduce the impact of frequency split on the gyroscope, we perform the frequency split compensation of gyroscope resonator through electrostatic negative stiffness effect mentioned above. The resonant frequencies of the gyroscope in the *X* and *Y* directions are extracted by two separate phase-locked loops, and closed controlled to keep the frequencies same. The residual frequency split of resonator is suppressed by applying appropriate direct current (DC) voltage to the excitation electrode. It is observed from the results that the frequency split of the resonator can be completely suppressed to zero, as shown in Figure 4.

### 3.2. Anti-Interference Design of External Environment

The external environment disturbance can be conducted into the gyroscope chip through the IMU, and then into the resonator, which brings the change of the internal physical structure of the resonator. The structure change possesses some randomness, which causes the nonlinear and unpredictable variation of installation error, scale factor error and ZERO error of the MEMS gyroscope. It directly leads to the loss of the MEMS IMU precision.

**Figure 5:**
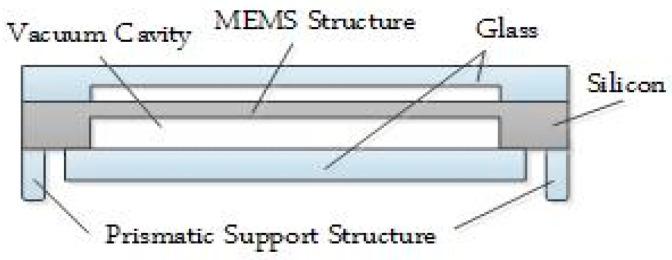
Structural design of the MEMS chip.

This paper proposes to set the bottom of the MEMS chip as a prismatic structure, as exhibited in Figure 5. This prismatic structure can decrease the effect of packaging stress, by reducing the contact area between the MEMS chip and substrate. At the same time, the vacuum cavity of the gyroscope chip is separated from the prismatic structure to limit the conduction path of external stress.

**Figure 6:**
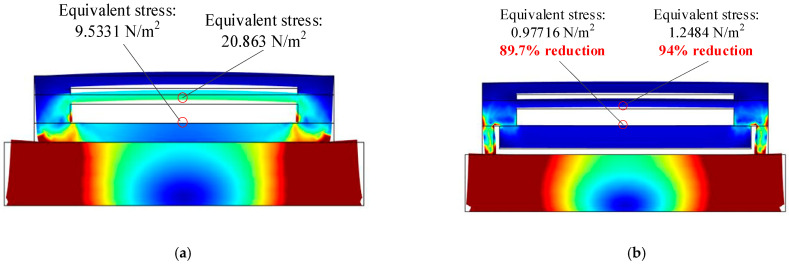
The simulation results of the equivalent stress between two kinds of chips: (**a**) the traditional MEMS chip; (**b**) the MEMS chip proposed in this paper.

The simulation results state that this prismatic structure at the bottom of the MEMS chip sufficiently lessens the conduction path from the external environment to the inside sensing structure on the resonator, enabling the equivalent stress of the resonator fine structure from external thermal environment to decline by more than 90%, as shown in Figure 6. 

## 4. Self-Calibration

Because of the adverse impacts of working environment and machining error, the zero position and scale factor of the MEMS gyroscope would be unstable. The traditional control mode could only calibrate and bind the zero position and scale factor of the gyroscope initially, while the real-time online calibration cannot be realized as time changes, which is severely detrimental to the precision and storage life of the gyroscope. 

This paper explores a real-time self-calibration method of MEMS gyroscope bias offset and scale factor based on dual control path, by which the resonance signal and high-frequency harmonic information of the gyroscope could be measured and controlled respectively with two controller modules with the same structure and function, thus obtaining the angle output information, zero position and scale factor information of the gyroscope in real time. Then, after the compensation for the data fusion of above information, the MEMS gyroscope can attain real-time online self-calibration.

**Figure 7:**
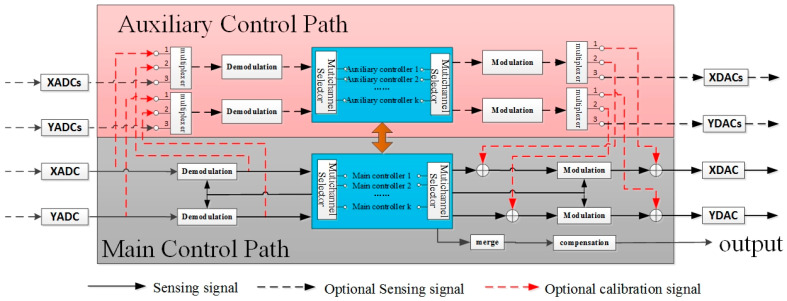
Schematic diagram of self-calibration algorithm.

The digital circuit mainly consists of the main control path and the auxiliary control path. Among them, the main control path is composed of the main controller, the orthogonal demodulation, and the orthogonal modulation module; the auxiliary control path includes the auxiliary controller, the auxiliary demodulation, and the auxiliary modulation module. Each control path contains an amplitude control loop, a phase-locked loop, an orthogonal control loop, a force equilibrium control loop, and so on, as indicated in Figure 7.

The signal processing circuit structure of the *X*-path and *Y*-path is identical (but the parameters can be different), so it can be reused to reduce the chip area. Nevertheless, the two paths are not simply corresponding to the driving and sensitive pathways of the traditional gyroscope chips.

The working condition of the whole system (such as working mode selection, on-off nature of the module, signal path selective switch, etc.) is controlled by the main controller. The auxiliary control path has the same circuit structure as the main control path, in which all the main modules are turned on or off depending on the working mode of the system. For example, when only the function of traditional gyroscope chip is needed, we could solely use the main control path, and the auxiliary control path can be turned off to ensure a lower power consumption level of the system.

The main controller and the auxiliary controller can work in different modes: for instance, the main controller is in closed-loop mode, though the auxiliary controller is in open-loop mode (or test mode, self-calibration mode, etc.). For example, for traditional asymmetric rate gyroscope, only one axis is suitable to be selected as the driving axis, and the other axis is taken as the sensitive axis, which is difficult to construct two gyroscopes with same working mode that have practical application value. At this moment, the main controller works in the traditional rate gyroscope mode (open-loop or closed-loop), while the auxiliary controller measures (open-loop) or controls (closed-loop) the harmonics of the drive shaft and sensitive shaft, whose harmonic information can be considered as a measurement of the nonlinearity degree of the system for self-calibration of scale factor or bias offset, or for nonlinear correction of the system. In other words, the main controller can be utilized to control and measure a MEMS gyroscope working on the resonant frequency, yet the auxiliary controller to control and measure a “virtual” gyroscope working on the resonant frequency of Nth harmonic. Afterwards, the information fusion of the two gyroscopes output could result in self-calibration, or improvement of some performance indexes (such as stability and repeatability).

The main controller and the auxiliary controller can exchange necessary information with each other. In fact, they can be regarded as the same controller. The parameters of the controller can be self-adapted to increase the adaptability of the system to external environment changes including the vibration, impact, pressure, humidity, and internal environment changes including temperature, stress, etc. The simultaneous information output from the main and auxiliary controller, which reflects the changes of external environment and internal parameters, can be combined and compensated to realize the self-calibration function, can be combined and compensated, so as to improve the repeatability and stability of the output.

## 5. Results

The actual size of the six-axis MEMS gyroscope unit is 72 × 72 × 35 mm^3^ and the weight is 210 g. Impressively, in the design of the IMU, the precision of the closed-loop controlled gyroscope could be further promoted by sacrificing the range and bandwidth for specific application situations. The research ideas are as follows: (1) To reduce the gain of the driving circuit: the proportional increase of digital control quantity when keeping the control objective unchanged could bring about the proportional increase of the scale factor. This way, however, would hamper the measurement range of the closed-loop gyroscope. (2) To lower the Proportion parameter of the Proportion Integration Differentiation (PID) controller: keeping the detection signal noise consistent could reduce the control quantity error, resulting in less error of digital control quantity, which, actually, is the gyroscope output signal. Nonetheless, this way would impair the gyroscope bandwidth. When the application environment needs of micro satellite have been satisfied, by the above methods (lifting the scale factor and lowering noise level), the gyroscope precision could be enhanced significantly at the acceptable expense of measurement range and bandwidth. In our study, the measurement range of the IMU is limited to 20 °/s, and the bandwidth to 12 Hz, to improve the precision index of the gyroscope.

**Figure 8:**
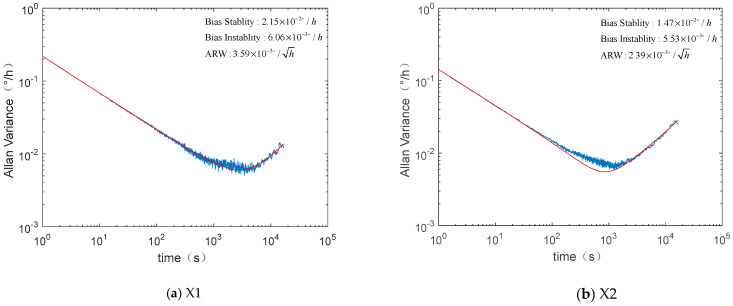

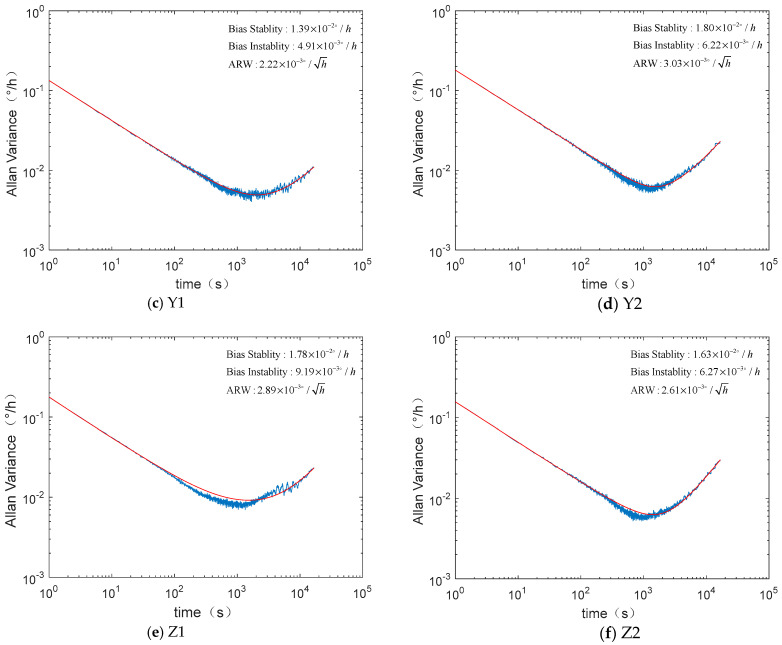
Allan variance curves of the six MEMS gyroscopes in the inertial measurement unit (IMU).

During the ground test, the IMU data were analyzed after continuous sampling for 24 h. It is summarized in Table 1 that the bias stability of the six gyroscopes in the MEMS IMU is between 0.01 and 0.02 °/h, with ARW of approximately 0.003 °/h^1/2^, and bias instability of about 0.006 °/h. Figure 8 shows the Allen variance of the six MEMS gyroscopes in the same unit.

**Figure 9:**
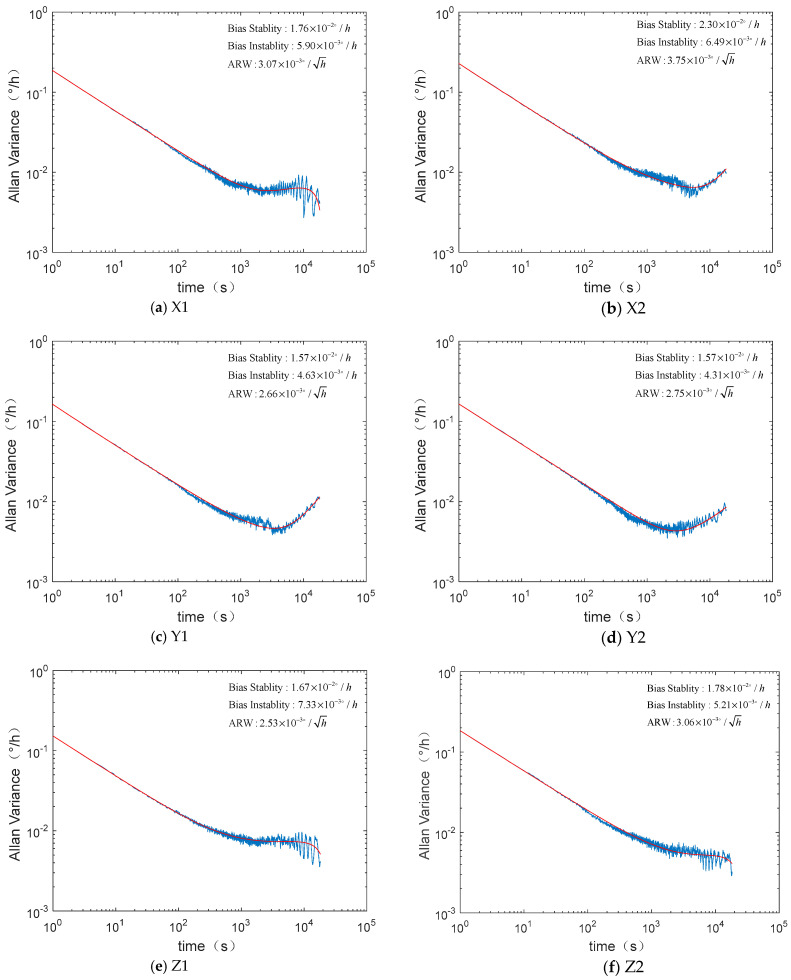
Allan variance curves of the six MEMS gyroscopes in the IMU tested 4 months later.

The same MEMS IMU was tested for another 24 h, about 4 months later. The data show that the bias offset of the gyroscopes were changed a little, as shown in Table 2, and the Allen variance of the six MEMS gyroscopes are shown in Figure 9.

**Figure 10:**
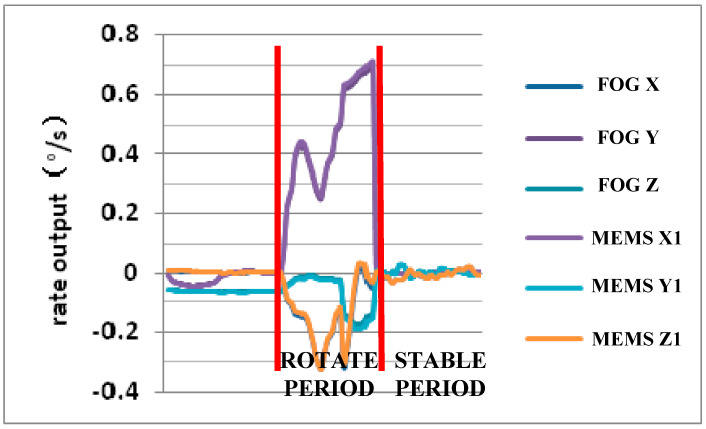
In-orbit data comparison between MEMS IMU and fiber optic gyroscope (FOG) unit.

The Lobster-Eye X-ray Satellite employed a MEMS IMU and a navigation grade fiber optic gyroscope (FOG) unit weighing 430 g. The in-orbit data of the MEMS unit transmitted from the satellite and the FOG unit almost completely overlap each other, as displayed in Figure 10, which means that the output and control effect of the MEMS IMU is similar to that of the FOG unit on the same satellite. Meanwhile, the weight and volume of the MEMS unit are only about 1/2 of the FOG unit.

## 6. Discussion

By sacrificing the range and bandwidth, the mechanical scale factor of the gyroscope could be promoted, which would further weaken the MEMS gyroscope noise. A six-axis MEMS gyroscope redundant IMU, that adopts double gyroscopes at one coaxial direction, is developed. Therefore, the performance of this MEMS unit can be further improved through data processing between the two gyroscopes at one coaxial direction. The performance of this six-axis MEMS gyroscope unit is shown in Table 3.

Furthermore, this product can directly replace the three MEMS gyroscopes in situ with three MEMS accelerometers, to turn it into a MEMS IMU with three MEMS gyroscopes and three MEMS accelerometers, which could be used for the high precise navigation.

## 7. Conclusions

The paper describes a new six-axis MEMS IMU with the bias stability of 0.02 °/h, bias instability of 0.006 °/h, and ARW of 0.003 °/h^1/2^, attaining the navigation grade, by means of customized gyroscope design and unique gyroscope self-calibration method. This product has been successfully put into use in the Lobster-Eye X-ray Satellite, which is the MEMS gyroscope commercial aerospace application with highest precision that has ever been reported worldwide. From the in-orbit data transmitted from the satellite, the performance of the MEMS IMU is competitive to the small FOG unit on the same satellite.

## 8. Patents

The following patents are resulted from the work reported in this manuscript: CN201310497005.5, CN201720895640.2

## Figures and Tables

**Table 1:** Ground test results of the inertial measurement unit (IMU).

Index	Unit	Performance					
		X1	X2	Y1	Y2	Z1	Z2
Bias offset	°/h	−13.49	−4.28	3.85	−12.35	−10.06	11.05
ARW	°/h^1/2^	3.59 × 10^−3^	2.39 × 10^−3^	2.22 × 10^−3^	3.03 × 10^−3^	2.89 × 10^−3^	2.61 × 10^−3^
Bias stability (1*σ*)	°/h	2.15 × 10^−2^	1.47 × 10^−2^	1.39 × 10^−2^	1.80 × 10^−2^	1.78 × 10^−2^	1.63 × 10^−2^
Bias instability	°/h	6.06 × 10^−3^	5.53 × 10^−3^	4.91 × 10^−3^	6.22 × 10^−3^	9.19 × 10^−3^	6.27 × 10^−3^

**Table 2:** Ground test results of the IMU 4 months later.

Index	Unit	Performance					
		X1	X2	Y1	Y2	Z1	Z2
Bias offset	°/h	−14.87	−2.01	3.79	−13.62	−9.34	11.03
ARW	°/h^1/2^	3.07 × 10^−3^	3.75 × 10^−3^	2.66 × 10^−3^	2.75 × 10^−3^	2.53 × 10^−3^	3.06 × 10^−3^
Bias stability(1σ)	°/h	1.76 × 10^−2^	2.30 × 10^−2^	1.57 × 10^−2^	1.57 × 10^−2^	1.67 × 10^−2^	1.78 × 10^−2^
Bias instability	°/h	5.90 × 10^−3^	6.49 × 10^−3^	4.63 × 10^−3^	4.31 × 10^−3^	7.33 × 10^−3^	5.21 × 10^−3^

**Table 3:** The IMU performance index.

Index	Unit	Performance
Range	°/s	±20
Bias Stability (1*σ*)	°/h	0.02
Bias Instability	°/h	0.006
ARW	°/h^1/2^	0.003
Bandwidth	Hz	12
Power	W	<1.5
Weight	g	210

## Data Availability

No New data were created or analyzed in this study. Data sharing is not applicable to this article.
